# Cognitive Behavioral Therapy for Three Patients with Bipolar II Disorder during Depressive Episodes

**DOI:** 10.1155/2020/3892024

**Published:** 2020-07-14

**Authors:** Yasuhiro Kimura, Sayo Hamatani, Kazuki Matsumoto, Eiji Shimizu

**Affiliations:** ^1^Department of Welfare Psychology, Faculty of Welfare, Fukushima College, Fukushima, Japan; ^2^Research Fellow of Japan Society for the Promotion of Science, Japan; ^3^Research Center for Child Mental Development, Chiba University, Chiba, Japan; ^4^Department of Cognitive Behavioral Physiology, Graduate School of Medicine, Chiba University, Chiba, Japan

## Abstract

Bipolar II disorder is a recurrent mental health disorder characterized by alternating hypomanic and depressive episodes. Providing cognitive behavioral therapy (CBT) as an adjuvant to pharmacotherapy can reduce the recurrence rate of bipolar disorder. It has not been examined whether CBT can be started during a depressive episode in patients with bipolar II disorder; however, the use of CBT during the remission period has been demonstrated to reduce recurrence. The current study is a case report involving three Japanese patients with bipolar II disorder, who started CBT during the depressive phase after a hypomanic episode was stabilized by pharmacotherapy. All patients experienced excessively positive thinking one week apart and were able to choose behaviors that would stabilize bipolar mood by observing its precursors. After intervention, patients' bipolar mood according to the Internal State Scale (ISS) and the Beck Depression Inventory-II (BDI-II) was improved. Our findings suggested that providing CBT to patients with bipolar II disorder during depressive episodes as an adjunct to pharmacotherapy is feasible.

## 1. Introduction

Bipolar disorder is a mental health disorder that is characterized by alternating manic and depressive episodes. It can occur in late adolescence or at any point in adulthood [1]. According to the World Mental Health Survey of Japan (WMHJ) conducted from 2002 to 2006 in 11 community populations, the lifetime prevalence of bipolar disease in Japan (including types I and II) is 0.2% [2]. Impairment of social functioning because of bipolar disorder is more severe than that caused by any chronic illness such as hypertension, diabetes, angina, heart failure, and arthritis [3, 4]. Furthermore, the impact on patients' families is heavy; a survey involving 226 families of patients with bipolar disorder showed that over 90% of families suffer from patient behavior problems [5]. Pharmacotherapy is the mainstay of the treatment of bipolar disorder, but this alone may not be sufficient, especially in drug-resistant patients [6]. A possible add-on to pharmacotherapy could be cognitive behavioral therapy (CBT), which consists of several sessions scheduled one week apart. A treatment time of 90 minutes or more has been suggested to be much more effective than a shorter treatment time [7]. This is probably because time-consuming discussions with the therapist provide an opportunity to patients to understand their illness and to learn and practice techniques that help them maintain their lives.

Regarding the effectiveness of CBT for bipolar disorder, studies have been recommending that this therapy be used as an adjuvant to pharmacotherapy to reduce the risk of recurrence and occurrence of depressive symptoms [7]. A moderate improvement of the severity of mania has been shown in a meta-analysis of 19 randomized controlled trials (RCTs) involving 1384 individuals. However, all 19 RCTs included in this meta-analysis have been conducted in the western cultural sphere such as UK, USA, Brazil, Spain, and Australia; no such studies were conducted in the East Asian countries such as Japan. As a typical CBT program for bipolar disorder, Lam's model of CBT was offered to such patients as an adjunct to pharmacotherapy and as a measure to prevent recurrence and efficiently monitor and coordinate their mood, thinking, and behavior [8]. Lam's model has been shown to have a remarkable preventive effect on relapse in patients after remission of bipolar disorder [8]; however, whether the adoption of this model is beneficial for patients during acute episodes of bipolar disorder has not been evaluated.

CBT for depression has been shown to be effective in improving symptoms, similar to pharmacotherapy [9, 10]. Therefore, patients with chronic persistent depressive mood can recognize their symptoms, mood, and behavioral patterns and actively reconstruct them into more functional patterns. Similarly, we hypothesized that patients with bipolar disorder can possibly understand cognitive behavioral models during the depressive episode and reconstruct their thoughts and patterns of behavior to maintain moderate mood.

Here, we present a case series involving three Japanese patients with bipolar II disorder, who were successfully treated with CBT based on Lam's model during a depression episode [8].

## 2. Case Presentation

### 2.1. Patients

Three patients, two women and one man, who met the *Diagnostic and Statistical Manual of Mental Disorders*, fifth edition (DSM-5) criteria for the diagnosis of bipolar II disorder, underwent individual CBT [1]. To distinguish them, the following fictitious names were assigned: “Kim” to patient 1, “Meg” to patient 2, and “Hank” to patient 3. Details on patients' background are presented in Table 1.  Table 2 shows hypomanic and depressive episodes. The authors assert that all procedures contributing to this work comply with the ethical standards of the relevant national and institutional committees on human experimentation and with the Helsinki Declaration of 1975, as revised in 2013 [11]. All patients were treated as part of a routine clinical service, and the project was considered a clinical audit. We explained the study thoroughly, and the patients signed the written consent form.

## 3. Medical History and CBT Introduction

### 3.1. Patient 1: Kim

Kim felt like her work partner denied her humanity one year ago. Kim consulted a psychiatrist after experiencing anxiety and forgetfulness. Two months later, after experiencing an improvement in mood, her mood fluctuated greatly between good and bad. She began to show hypomanic behavior (e.g., slept a few hours and extravagance). On the contrary, when she was feeling down, she reportedly cried uncontrollably, lost her appetite, and often had thoughts of worthlessness. Eventually, Kim was unable to go to work and consequently took time off. Bipolar mood was alleviated by mood stabilizers and time off, but self-responsible thinking and depressed mood afflicted her. Upon her psychiatrist's recommendation, she agreed to undergo psychotherapy and visited the university to which the lead author is affiliated. The main diagnosis at this time was “bipolar II disorder,” which corresponded to a hypomanic episode according to the Japanese version of the Simple Structured Interview for Mental Illness (M.I.N.I) [12, 13].

### 3.2. Patient 2: Meg

After exhibiting frequent rude behavior with an employer, experiencing depressed mood and remorse, and temporarily having to take sick leave, Meg was diagnosed with depression at an approximate age of 40 years. Since then, she experienced dramatic mood fluctuations and was diagnosed with bipolar II disorder. Meg was able to work again and continue her job with regular visits to hospital and pharmacotherapy until retirement; however, she endured bipolar episodes several times a year. After enduring several hypomanic episodes in year X, Meg decided to undergo cognitive behavioral therapy, recommended by her family and physicians.

### 3.3. Patient 3: Hank

Until the age of 20 years, Hank lived with his father (who probably had bipolar disorder), mother, and brother. After his parents' divorce, Hank entered college and moved away from his father; at the age of 24 years, after graduating, he started working and felt that it was worthwhile. When he was 25 years old, his mother died of heart disease, after which Hank and his older brother lived separately. Hank began to work harder to counteract lonesomeness. When he was 26 years old, he was dismissed from his job because of ulcerative colitis, probably caused by overworking. Even after returning to work, Hank repeatedly accepted more work than he could manage: later he recalled that “I had probably been repeated episodes of hypomania and depression.” At the age of 27 years, Hank frequently took breaks from work because of marked depression; after being referred to a psychiatrist, he was diagnosed with “bipolar type II disorder.” Hank received pharmacotherapy, but he was unable to realize its effect and took a leave of absence on job. At the age of 29 years, Hank checked for bipolar disorder on several websites, hoping to receive a CBT program. Hank was referred to the second (SH) and the last author (ES).

## 4. Measures

Participants' mood during daily life activities was assessed at the first and last CBT sessions to evaluate the effectiveness of treatment. Key results were measured using the Beck Depression Inventory-II (BDI-II). BDI-II can help the quick assessment of the severity of depressive symptoms; it includes 21 questions about the status of mood during the past two weeks (scores: 0–13, minimal; 14–19, mild; 20–28, moderate; and 29–63, severe) [14, 15]. The Internal State Scale (ISS), a 16-item self-written 100 mm visual and analog scale, was used to assess the severity of manic and depression symptoms [16–18]. The ISS has four subscales: activation, perceived conflict, well-being, and depression. The standard scores for the clinical status of ISS are as follows: well‐being < 125 for depressive symptoms and >125 for mania and activation > 200.

## 5. Intervention (Cognitive Behavioral Therapy)

Individual face-to-face CBT was provided once a week. Kim attended seven sessions, Meg attended 10 sessions, and Hank attended 16 sessions, and each session lasted for 50 minutes. CBT consisted of the following six modules. The patients in this study did not need 90 minutes because they were actively involved in the treatment. Hank took more time to learn CBT skills than other patients, so he had undergone more sessions.

### 5.1. Psychoeducation and CBT for Bipolar Disorder

In the context of a stress vulnerability model, behavioral activation, reward responses, kindling phenomena, circadian rhythm disorders, and dysfunctional beliefs in patients with bipolar disorder are described. With regard to behavioral activation and reward response, patients with bipolar disease who are hypersensitive to emotions are explained that persons with bipolar disorder often feel positive emotions, which can enhance elation, increase purpose-directed activity, and decrease sleep needs, reckless behavior, instability, and anger [19]. Patients were provided with the knowledge that active behavioral systems make them have a shorter relapse time [20]. Kindling, a “persistent and possibly permanent neuroexcitable change,” was also introduced, explaining that later episodes of mania rather than the first one could be caused even if there is less stress [21].

### 5.2. Case Conceptualization and Therapeutic Goal Setting

**Figure 1:**
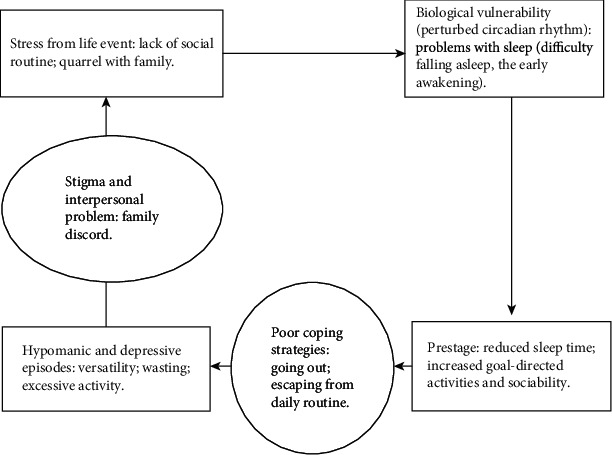
Patient 2 Meg's case formulation.

Normally, the patient undergoing CBT becomes aware of the conventional vicious cycle of excessive activation and depression by performing case formulation, and therefore, he/she can select a more appropriate behavior. The therapist helped the patients formulate his/her condition at all times after treatment (Figure 1, patient 2's vicious cycle). It is known that risk factors include high-effort goals, goal orientation, and the dysfunctional belief of perfectionism [22]. The therapist and patient discuss acquiring more adaptive cognitive and behavioral patterns to better spend their lives in the long term. Therefore, setting therapeutic goals is a crucial factor early in the treatment. By encouraging goal-oriented behavior to be achieved from a longer-term perspective, patients can be motivated to try to control their commitment to excessive activity.

### 5.3. Monitoring Activation and Mood

Most patients with bipolar disorder complain of prodromal symptoms, and the pattern of prodromal symptoms is fairly consistent [8, 23, 24]. In patients with bipolar disorder, a dysfunctional belief in achieving goals is elicited after a positive mood [25]. If patients with bipolar disorder monitor their perceptions of positive moods, beliefs, and prodromes, it might be possible to help them control further mood upliftment. Patients can acquire skills to identify mood and associated activities using self-filled symptom assessment scales, mood diaries, and mood observation forms [26].

### 5.4. Mastering Coping Behaviors with Prodromes of Hypomanic and Depressive Episodes

Evidence suggests that patients respond appropriately to prodromal symptoms to improve social functioning and to extend the time to the next hypomanic episode [23, 27]. To prevent progression to a complete hypomanic episode, the therapist should suppress goal-directed behavior during the progenitor phase of the hypomanic phase and not be still during the prodrome of depression to lead to a regular life [26].

### 5.5. Establishing Sleep and Daily Routine

Sleep and wakefulness have been shown to be important in both hypomanic and depressive episodes [28, 29]; explaining the significance of establishing a sleep habit to patients is important in preventing recurrence. If sleep and routine are irregular, circadian rhythms are more likely to be disrupted, and this can cause progression to hypomanic episodes [30, 31]. Because human circadian rhythms are synchronized with social events and daily routines, minimizing disruptions in patients' circadian rhythms is important to help patients establish adaptive social routines [26]. The patients established sleep and daily routine with behavioral skills such as activity schedules.

### 5.6. Rescripting Dysfunctional Beliefs

Patients with bipolar disorder have a strong motivation to achieve their goals; assumptions such as the following are typically observed: “If I work hard, I should be able to do everything better than others,” and “I have to do it well [32].” The desire for success makes them to work for extended periods of time and ignore important social routines, such as regular meals and exercise [26]. By cognitive reconstruction and identification of beliefs and revising them to more adaptive beliefs, patients can rescript their patterns of cognition and behavior.

## 6. Course of Treatment

**Figure 2:**
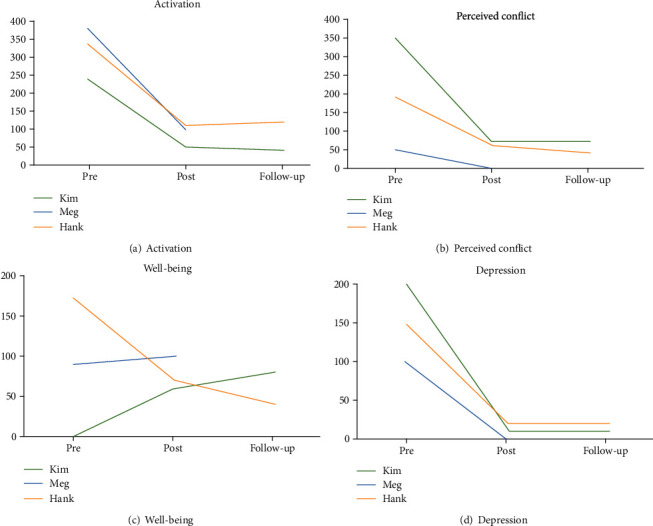
Change of subscales in ISS scores.

All patients completed the cognitive behavioral therapy module (see Table 3 for details).  Figure 2 shows the changes in the evaluation items, BDI-II and ISS, before and after the session.

### 6.1. Patient 1: Kim

Seven sessions of therapy, 50 minutes each, were conducted. The last session was a follow-up session, held 18 months after the completion of the first 6 sessions. Although Kim experienced self-critical thoughts and negative effects (e.g., depression and anxiety) with high frequency, she performed few coping-related activities to change her mood. Therefore, we hypothesized that this might be because she could not accurately grasp her mood state. It was also confirmed that increased stress-associated loss of appetite and sleep were her prodromes. She was asked to record her mood state on a scale of 1 to 100 and to record how it changed after activities. As a result, she became more aware of her negative mood; she began coping with negative effects by performing various activities (e.g., talking with a friend, reading, and looking at her favorite pictures). She said that her emotions were easier to control than before. She also had to talk to her employer regarding returning to work, but she avoided her appointment because she thought, “Will my request to return be denied if I go to the meeting?” We tried to deal with this problem by using cognitive restructuring. She was able to make an appointment herself and to have an interview with her employer by thinking, “I am thinking too much. I might get different results if I try.”

The BDI-II score decreased from 27 (before the intervention) to 7 (after the intervention). One month later, this score decreased further to 2. The score remained at 2 points even during the follow-up. With regard to ISS, the activation score improved from 240 to 50, the conflict score from 350 to 70, the well-being score from 0 to 60, and the depression score from 200 to 10. At follow-up, activation, conflict, well-being, and depression scores were 40, 70, 80, and 10, respectively; all scores remained stable until follow-up. During the follow-up session, she told that she had changed jobs and that she got married. She reported that she continued to control her mood by being aware of her mood state. Furthermore, she placed the therapy material in a place where she can always see it. When she experienced stress, she would reread it and increase her ability to cope with stress.

### 6.2. Patient 2: Meg

Early in the interview, Meg prepared a case conceptual diagram with her therapist (Figure 2). Meg and the therapist hypothesized that the trigger for going out was a cluttered home environment and decided that disposing clothes that she would no longer wear was a solution, during the session. The daily complaints from her husband and daughter about cluttered clothes were very stressful for Meg. Meg and the therapist set homework to dispose of one clothing item every day from her home. During ten sessions, Meg disposed of 200 clothing items and tackled other housework such as cleaning a bass and the toilet and washing dishes. The clean and calm rooms made Meg more comfortable and feel a lesser need to go out and lesser sense of being wasted. Meg was delighted to have her family praise her homework and housekeeping. During the middle stage of CBT, we used a habit activity monitoring sheet to conduct training and to notice and deal with precursor symptoms. Meg found that she was relatively calm and did not shop impulsively, if she met her friends. Fortunately, Meg had so many friends. Meg decided to invite friends a couple of times a week and talk for a few hours, to prevent mood uplift and live a calm life. At the end of CBT, the precursor symptoms for Meg (going out, shopping, lunch, learning, and traveling) were identified, and the specific measures to be taken when the mood is disrupted (cleaning up, doing housework, standing still, going to an ink painting classroom, and having tea) were listed on a coping card. The coping card had been placed in Meg's wallet, and she read them aloud every day.

With regard to symptom changes before and after the treatment, the BDI-II score for depressive symptoms dropped from 22 to 4. With regard to ISS, the activation score of the ISS improved from 380 to 100, the conflict score from 50 to 0, the depression score from 100 to 0, and the well-being score from 90 to 100. Meg expressed that she was happy with the results of these scales and her daily life saying “I have not felt so stable in the last 20 years. Everything is better.”

### 6.3. Patient 3: Hank

The first CBT session was conducted while continuing with pharmacotherapy; we monitored mood to notice prodromal symptoms. In the third session, the medication was changed (Lamictal 200 mg); it was maintained thereafter. He noticed irritability, being more vocal, working longer, shopping more, and sleeping less as precursor symptoms; he also noticed a disruption in his rhythm of life. Moreover, when he was depressed, he laid down and slept longer and did nothing all day. When the room was dirty, he felt lonely and depressed; therefore, he chose to do household work for about 10 minutes every day. In addition, he was absent from work when he felt stomachache, spending a day playing games at home while resting. However, it was clear that his physical condition would not recover, and depressive mood would appear and aggravate with “I am so useless.” Therefore, we planned to perform the following routines regardless of his mood: decide when to get up, go to work, do 10 minutes of household work, do muscle training, and go out on either Saturday or Sunday. Furthermore, when he suddenly wanted to shop or do any other activities, he decided to buy after one week, not immediately, or postponed the action. Furthermore, Hank hesitated to contact his brother by himself, because he and his brother are estranged, and he thought that his brother would not be pleased. However, Hank contacted his brother to find out if it was true; he stated it was fun to meet him and began to meet him regularly.

The effect of treatment was remarkable, and the symptom scale score changed. Hank reported a more stable mood and a better quality of life. “I was basically depressed from around the age of 20, but now I can go to work and live with friends over the weekend, regardless of my mood.” The BDI-II score for depressive symptoms decreased from 41 to 5. With regard to ISS, the activation score changed from 340 to 110, the conflict score from 190 to 60, the depression score from 150 to 20, and the well-being score from 170 to 40. There was no recurrence at a 6-month follow-up: BDI-II score, 10; ISS activation, 120; conflict, 40; depression, 20; and well-being, 40.

## 7. Discussion

This article examined the feasibility of providing personal cognitive behavioral therapy based on Lam's model to three Japanese patients with bipolar II disorder during a depressive episode [8]. No adverse events were reported during CBT. All patients were able to complete the CBT modules. Judging from the BDI-II and ISS scores before and after the treatment, CBT resulted in reduced symptoms of hypomania and depression. The results of this study show for the first time that the CBT based on Lam's model is feasible for Japanese bipolar II disorder in the world.

This study showed promising results for the long-term prognosis of patients with bipolar disorder, which are consistent with those of previous studies [45]. According to a previous meta-analysis that included eight RCTs, when cognitive behavioral therapy was provided as an adjuvant to pharmacotherapy to patients after remission, the recurrence rate was about 40% lower than that with pharmacotherapy alone (odds ratio 0.53; 95% CI, 0.39–0.72; *p* = 0.001) [46]. In the present study, no patient of two patients relapsed at the follow-up 8 months or 18 months after the end of the intervention. A previous study on the prevention of recurrence of bipolar disorders has studied the effects of Lam's CBT model in patients without episodes, including RCT designs (1 year). It has been reported that pharmacotherapy plus CBT for patients with bipolar disorder reduces the rate of subsequent recurrence (odd recurrence rate: 44% in the pharmacotherapy plus CBT group; 75% in the pharmacotherapy-alone group) [45].

In patients with bipolar disorder, CBT based on Lam's model targets the distorted cognitive state called “hyperpositive thinking (excessive optimism, extreme self-confidence, and risk underestimation) [8].” With hyperpositive thinking, patients with bipolar disorder exaggerate their dynamism, persuasiveness, and productiveness, and consequently, they increase their activity. Because such activity can sometimes be a method of avoidance or escape from negative emotional states, patients would have more serious problems related to social function. Lam's model of cognitive behavioral therapy is offered to such patients as an adjunct to pharmacotherapy and as a measure to prevent recurrence and efficiently monitor and coordinate their mood, thinking, and behavior [8]. Lam's model has expanded on the findings of circadian rhythm instability as a biological vulnerability inherent in bipolar disorder [47], added disruption of daily routine, and lack of sleep due to stress factors such as life events. Lam's model has been shown to have a remarkable preventive effect on relapse in patients after remission of bipolar disorder [8], and the importance that patients prevent relapse of manic episode by keeping sleep and daily routine has emphasized. Our results suggest evidence that CBT for bipolar disorder can be initiated during a depressive episode [48]. Bipolar disorder is complex and requires treatment at various stages, because pharmacotherapy or psychotherapy depends on the ability of the therapist to select problems to be treated at a particular stage of the disease [49]. Thus, the findings from this study of the timing of initiation of psychological intervention programs expand Lam's model, because the model is generally implemented during remission in bipolar disorder [26].

The primary aim of using CBT for bipolar disorder is to stabilize mood and behavior [26], and patients need to acquire the skills to grasp mood fluctuations. If the patients identify the mood fluctuations by themselves, they could notice the precursor symptoms and employ coping mechanisms. In this study, we provided cognitive behavioral therapy to Japanese patients with bipolar II disorder during a depressive episode, and by using prognostic symptoms and using cognitive behavioral skills, patients were able to calm episodes of depression and hypomania. Patient 1 (Kim) was able to catch prodromal symptoms and take more appropriate actions based on psychological education. Although other patients understood “hyperpositive thinking,” they took time to transform thinking and behavior that helped them overcome the problem. However, they had family conflict or loneliness as a barrier. Since patient 2 (Meg) and patient 3 (Hank) had received problem-solving training, they proved to be able to address them. During the course of treatment in this study, family dysfunction was seen in two cases, which appeared to be related to a hypomanic episode. A previous study suggested that bipolar disorder is associated with dysfunction in families and conflict in families [50]. Another study also suggested that significantly less active and positive engagement and communication between siblings within families are associated with bipolar disorder [51]. Restoring family function may help stabilize symptoms and prevent recurrence, and our results suggested that clinicians can provide patients with other cognitive behavioral skills, such as problem-solving techniques, if family dysfunction is significant. Patient 2 (Meg) improved family relationships by disposing clothes, and patient 3 (Hank) eased loneliness through reunion with a distant older brother. Family relationships were improved, and Meg and Hank could focus on their problems of hypomanic and depressive mood more.

Finally, the findings of this study have to be seen in light of some limitations. This study cannot claim certain effectiveness, because the series included only three case studies. To examine the efficacy of the CBT model for patients with bipolar II disorder in the future, it is necessary to conduct clinical trials designed to include random controls. In the future, a randomized controlled trial with a larger sample size and a control group should be conducted to verify efficacy. In addition, when initiating an intervention during a period of depression, care must be taken to distinguish between symptomatic improvement and hypomanic switch. As countermeasures against this problem, the use of multiple evaluation tools and the collection of information from third parties such as family members can be considered. However, in this report, the fact that evaluation is conducted only by the ISS is a limitation.

## 8. Conclusion

We found that patients with bipolar II disorder could possibly cope with episodes of depression when Lam's model-based CBT is used to help them recognize the precursors of their own hypomanic episode. The results of this study suggest that depression episodes in patients with bipolar II disorder are a good time to start Lam's model-based CBT. CBT as an adjunct to pharmacotherapy can be probably beneficial in enhancing stabilization for patients with bipolar II disorder [52, 53].

## Figures and Tables

**Table 1:** Patients' background and characteristic.

	Kim	Meg	Hank
Sex	Female	Female	Male
Age	24 years old	64 years old	29 years old
Age of onset	23 years old	40 years old	20 years old
Marital status	Single	Married	Single
Education level	College	Junior college	College
Employment status	Nursey teacher	Maturity retirement at a nursery teacher	Programmer
Comorbidity	None	None	None
Pharmacotherapy	Lithium carbonate 400 mg, blonanserin 1 mg	Lithium carbonate 200 mg	Lamotrigine 200 mg

**Table 2:** Patients' mood episode.

	Hypomanic episode	Depressive episode
Kim	23 years old winter	24 years old spring
Meg	40 years old winter	41 years old spring
	62 years old spring-autumn	64 years old spring
Hank	20 years old spring	24-25 years old
	25-26 years old	27 years old summer

**Table 3:** Tasks and contents of each patient.

Module	Patient 1 (Kim)	Patient 2 (Meg)	Patient 3 (Hank)
A. Psychoeducation	Patients were provided with the following information: the prevalence of bipolar disorders is about 2% and the prevalence in men and women, which reaches 5% when subthreshold bipolar conditions are included, is about the same [33, 34]. The highest risk is associated with adolescence, especially peaking between the ages of 15 and 20 [34, 35]. Bipolar disorder is prone to significant social disability and to relapse [36, 37]. It is also reported that episodes of mania and depression are periodic [38, 39] and that episodic periods can become increasingly shorter [40]. Anxiety plays a significant role as a risk of recurrence [41], and there is a tendency for worse outcomes in the presence of substance abuse [42, 43]. Suicide rates are 0.4% per year, 20 times the rate of suicide in the general population [39]. Furthermore, patients were introduced to the six most common precursors to mania (decreased sleep time, increased goal-directed activity, increased irritability, increased sociability, lost thought, and increased optimistic thinking) [23, 24, 44], and they were explained that being aware of his prodromal symptoms and developing and practicing skills to cope are the core of CBT [26].		
B. Case conceptualization and therapeutic goal setting	When she meets a person, she thinks “Is she going to say something negative?” She reinforces the belief, “I'm worthless.” As a result, she does not leave the house and does not consult anyone. Goals: increase outings, resume hobbies, and talk to colleagues.	Meg was depressed about the fact that the inside of the house is scattered, and the nonfunctional belief, “I am lazy and useless,” is activated. Meg drives purpose-oriented activities to regain self-confidence; therefore, she always postpones annoying routines. Goals: cleaning up her house, managing money, and keeping on living comfortably without bipolar disorder.	Hank's mood is uplifting; he overdoes his work, makes mistakes, and activates the nonfunctional belief, “I am a bad person.” To get away from it though, he was immersed in work. Goals: determine the amount of work, manage money, and join the community.
C. Monitoring activation and mood	Hypomanic: decreased sleep time, increased activity, talkativeness, and extravagance. Depression: loss of appetite, self-responsibility, worthlessness, loss of interest, abdominal pain, and headache.	Hypomanic: decreased sleep time, increased purpose-oriented activities, increased sociability, increased optimistic thinking, and talkativeness. Depression: decreased interest and activity, sad mood, reduced motivation, reduced self-esteem, and pessimistic thinking.	Hypomanic: reduced sleep time, increased activity, talkativeness, frustration, increased money, increased work, and approaching women. Depression: abdominal pain, decreased interest and activity, sad mood, reduced motivation, pessimistic thinking, and increased sleep.
D. Mastering coping behaviors with prodromes	Hypomanic precursors: taking a break, calling a family member, and consulting a physician. Depression precursor: meeting friends and family and watching favorite pictures.	Hypomanic precursors: doing daily routines such as housekeeping and cleaning and performing unscheduled actions (delayed) in the 24-hour transition. Depression precursor: exercising and meeting friends.	Hypomanic precursor: when shopping, do not buy immediately. Wait for a week, before deciding to buy things added to an online shopping basket. Depression precursor: walking, meeting an older brother, not falling asleep, and not reflecting alone.
E. Establishing sleep and daily routine	Schedule activities during the day, wake up on time, and consume meals regularly.	Meg's daytime activity has improved her sleep quality at night. She refrained from taking a nap and excessive caffeine.	Hank decides to go to work on weekdays, even if he does not feel good. Overtime is restricted to 19:00, even if there is work left. Hank managed bathing, muscle training, and game time.
F. Rescripting dysfunctional beliefs	Kim identified the beliefs “I am worthless” and “Everything will fail” and rewrote it as “The future is unpredictable, let us act first.”	Meg identified the belief “I am useless and worthless” and rewrote it as “I am loved by friends and family.”	Hank identified the belief “I am a bad person and I will lonely for life” and rewrote it as “I am single now; I do not know what will happen in the future. I have brothers and friends.”
